# Use of large-scale HRQoL datasets to generate individualised predictions and inform patients about the likely benefit of surgery

**DOI:** 10.1007/s11136-017-1599-0

**Published:** 2017-05-31

**Authors:** Nils Gutacker, Andrew Street

**Affiliations:** Centre for Health Economics, University of York, Heslington, YO10 5DD UK

## Abstract

**Purpose:**

The English NHS has mandated the routine collection of health-related quality of life (HRQoL) data before and after surgery, giving prospective patient information about the likely benefit of surgery. Yet, the information is difficult to access and interpret because it is not presented in a lay-friendly format and does not reflect patients’ individual circumstances. We set out a methodology to generate personalised information to help patients make informed decisions.

**Methods:**

We used anonymised, pre- and postoperative EuroQol-5D-3L (EQ-5D) data for over 490,000 English NHS patients who underwent primary hip or knee replacement surgery or groin hernia repair between April 2009 and March 2016. We estimated linear regression models to relate changes in EQ-5D utility scores to patients’ own assessment of the success of surgery, and calculated from that minimally important differences for health improvements/deteriorations. Classification tree analysis was used to develop algorithms that sort patients into homogeneous groups that best predict postoperative EQ-5D utility scores.

**Results:**

Patients were classified into between 55 (hip replacement) to 60 (hernia repair) homogeneous groups. The classifications explained between 14 and 27% of variation in postoperative EQ-5D utility score.

**Conclusions:**

Patients are heterogeneous in their expected benefit from surgery, and decision aids should reflect this. Large administrative datasets on HRQoL can be used to generate the required individualised predictions to inform patients.

## Introduction

“But will this treatment help *me*?” This simple question reflects one of the most commonly voiced concerns in many consultations with a doctor. Patients facing surgery have always wanted to know about the risks they face and whether treatment will be effective. Nowadays patients increasingly want to be actively engaged in the (co-)management of their medical condition, including the choice of treatment. To be able to participate in shared decision-making (SDM) patients require information on the relative effectiveness of alternative treatment options. But the effectiveness of medical treatments is often moderated by patient characteristics, such as age, gender, co-morbidity burden or genetic factors [17]. Hence, for information to be most relevant for the specific SDM context, it needs to reflect patients’ personal circumstances closely [1].

Randomised controlled trials, which are seen as the gold standard in effectiveness research, assess the average effectiveness across the study population. This information is, of course, most useful to prospective patients who share the same characteristics of the average person enrolled in the trial. But patients enrolled in trials tend to be systematically different from those to whom treatment will be given in routine practice and, of course, all patients are different. In recognition of this, there is rapidly growing literature on risk stratification and the concept of personalised medicine [2, 15, 25, 26]. The aim is to distinguish different groups of patients according to their observable pre-treatment characteristics so as to derive personalised predictions of their expected outcomes that are, *ceteris paribus*, more targeted than those based on experiences of the average patient who has previously had the treatment. However, these developments have not yet found their way into many popular decision aids used in routine clinical practice. In part, this may reflect the lack of sufficiently large medical studies that allow for fine-grained subgroup analysis. Even those trials that are powered for subgroup analysis tend to focus only on a limited number of single-factor contrasts. They are not, therefore, suitable for generating detailed risk profiles.

The emergence of large, routinely collected longitudinal datasets on patients’ health-related quality of life (HRQoL) opens up the possibility to move away from exclusive focus on average experience and to develop detailed risk stratification models. Since April 2009, the English NHS has mandated the routine collection of patient-reported outcome measures (PROMs) from all NHS-funded patients undergoing planned hip or knee replacement, varicose vein surgery or groin hernia repair. Patients are asked to report their health status and HRQoL using the EuroQol-5D-3L (EQ-5D-3L) and condition-specific instruments before and some months after surgery. By March 2015, over 800,000 patients had participated in these surveys and reported pre- and postoperative health measures. These data can be used for the purpose of risk stratification.

The aim of this paper is to report on the development of an online patient information tool (http://www.aftermysurgery.org.uk) and the underlying algorithm that utilise this large amount of HRQoL data to generate personalised (i.e. risk stratified) predictions. This tool is designed to be used by patients in consultation with their primary care physicians and general practitioners (GPs) in discussions about the likely benefits of surgery. The format of the tool draws on recent literature on the most suitable presentational format of HRQoL data to inform patients and medical professionals. In what follows, we describe the data and the analytical approach to risk stratification. We then describe how the tool has been developed and piloted, and provide examples of its presentational form. We conclude by outlining the next steps in its development and rollout for use to inform SDM between patients and their doctors.

## Methods

### Data

We utilise individual-level EQ-5D-3L data on all NHS-funded patients in England aged 15 or over who underwent planned unilateral hip or knee replacement or groin hernia repair between April 2009 and March 2016 [5].^1^ Patients are invited to report their HRQoL using paper-based questionnaires at two time points: at the time of admission or in the preceding outpatient appointment, and then again three months after surgery (6 months for orthopaedic procedures); see [10] for full details on data collection. These data are anonymised and made publicly available by the Health & Social Care Information Centre (HSCIC) (http://www.hscic.gov.uk/proms) and form the basis of our risk stratification algorithm. Patients were excluded if they underwent revision surgery or if relevant data items were missing (complete case analysis). Data released prior to the financial year 2012/2013 did not distinguish between primary and revision joint surgery. We therefore obtained individual-level EQ-5D-3L data linked to administrative hospital records (Hospital Episode Statistics) for these financial years to reconstruct the necessary revision flag from OPCS 4.6 procedure codes [5] and then applied the HSCIC anonymization rules.

The EQ-5D-3L measures health-related quality of life along five health dimensions [3]: mobility, self-care, usual activities, pain and discomfort, and anxiety and depression. On each dimension, patients can indicate whether they have no, some or extreme problems. The resulting health profiles are summarised using utility weights obtained from members of the general public in England [6], anchored at 1 (full health) and 0 (dead), with scores <0 indicating states worse than being dead. In addition, the dataset contains information on patients’ age (in 10-year bands), sex, self-reported duration of symptoms, and self-reported co-morbid diagnoses (high blood pressure, stroke, diabetes, poor circulation, depression, arthritis, cancer and diseases of the lung, liver, heart, kidneys, or the nervous system). Furthermore, patients indicated their overall assessment of the outcome of surgery on a five-point scale (*‘Overall, how are your [hip/knee/hernia] problems now, compared to before the operation?’* with answers *‘much better’*, *‘a little better’*, *‘about the same’*, *‘a little worse’*, *‘much worse’*).

No ethical approval was required for analysis of anonymised secondary data.

### Risk stratification

The aim of our empirical analysis was to generate algorithms to allocate prospective patients to strata or groups of similar expected postoperative utility scores. We employed non-parametric data mining techniques to populate separate regression trees for each of the treatments [17, 30]. The trees were generated through a recursive Classification and Regression Tree (CART) algorithm that split the dataset along risk variables to generate nodes and then repeated this process for each resulting tree branch until the dataset could not be split further or the overall fit of the model could no longer be improved. The resulting tree branches represent conjunctions of patient characteristics, and each branch ends in a strata allocation (‘leaf’). Patients within a strata have similar expected outcomes, but their realised outcomes may differ due to random variation or unmeasured determinants. This uncertainty is reflected in the distribution of observed outcomes within a strata.

Our candidate set of split variables included all pre-operative patient characteristics available in the dataset. However, after discussions with GP stakeholders and patients, it was decided that a limit on the number of variables needed to be imposed so that the tool could be used within a typical 10-minute doctor consultation. Exploratory analysis revealed that only few self-reported comorbidities led to branch splits and only in few instances. The final set of risk variables thus included only age, gender, pre-operative EQ-5D-3L profile and symptom duration, this limited set offering a balance between parsimony and explanatory power. Patients reporting health profiles of 11111 or 33333 prior to surgery were analysed separately and subsequently added to the classification algorithm. Patients in these pre-operative health states cannot improve/deteriorate but, due to the low frequency, may have been included erroneously within other groups had they not been analysed separately. This would otherwise have created logical inconsistencies in the presentation of results (see below) for these patients.

All analyses were performed in R3.2.1 using the CART package. The advantage of CART analysis over a more traditional regression analysis lies in the way the former handles interactions between variables and non-linearities. By considering all possible variable splits and orderings, and only retaining the model that fits the data best, CART identifies all relevant interactions and can easily incorporate non-linear effects of continuous or categorical variables. However, this data-driven modelling approach may lead to overfitting and poor predictive ability in independent samples. Overfitting occurs if *“idiosyncracies in the data are fitted rather than generalizable patterns”* ([28], p. 5). Since the structure of the statistical model is uncertain, the flexibility granted to the CART algorithm can result in a statistical model (here: grouping) that fits the data at hand but is less informative or potentially misleading to future users. To explore this, we used all data up until March 2015 (development sample) to estimate the regression trees and then calculated the model fit in terms of adjusted $R^{2}$ and root mean squared error (RMSE) in a sample of patients treated between April 2015 and March 2016 (test sample), where we include indicator variables for each of the strata.

### Presentation

For the information presented in the online tool to be useful to patients and their GPs, it needs to be easily interpretable and meaningful and not overburden the recipient with detail [11, 23]. A large literature has explored how best to communicate information to patients, and a recent series of studies focussed on patients’ and doctors’ preferences for and ability to interpret different presentational formats of hospital performance information based on HRQoL data [12–14]. Many of their findings apply to presentation of HRQoL data more broadly and have informed this work.

#### Content

An important conceptual choice in the development of our patient information tool has been between focussing on either the change in HRQoL as a result of treatment or the postoperative level of HRQoL. Both approaches have merit and convey important information. Patients are naturally interested in whether treatment improves their HRQoL given their individual starting points, i.e. whether treatment is effective. At the same time, understanding the absolute level of health they are likely to achieve may facilitate comprehending the potential benefits in terms of patients’ ability to participate in everyday life, and may also lead to more realistic expectations. Treatment may well improve their HRQoL but not restore them to a level that they regard as sufficient to warrant surgery (and associated risks). For the purpose of this patient information tool, both types of information are therefore presented.

#### Metrics

A closely related question is then how to make these data meaningful to the recipients. PROM scores are unfamiliar to patients (and often doctors as well) and *“unlike measures of height or weight,* [$\ldots$] *their values have no immediate meaning. It’s therefore necessary to transform them into interpretable forms, or indeed into experiences rather than metrics, to make them useful”* ([14], p. 11).

For measures of change one metric that has been advocated is the ‘minimally important difference’ (MID). The MID can be derived in a number of ways. We followed the anchor-based methodology employed recently by [4] to obtain MIDs for our study sample.^2^ The MID for improvements is calculated as the difference in EQ-5D utility change score between all patients that reported their problems as ‘a little better’ and those that report their problems as ‘about the same’. The MID for deteriorations is calculated in a similar way. Different MIDs are calculated for each of the three procedures. We then calculate the proportion of patients in each strata that have noticeably improved, did not experience a noticeable change, or have noticeably deteriorated.

For postoperative levels, we report the proportions of patients reporting no/some/extreme problems by EQ-5D dimension.

#### Format

Concerns have been voiced about patients’ ability to interpret numeric information and different presentational formats. Pictographic presentation of data is generally well understood and accepted and has been advocated for risk communication [8, 12, 24, 29]. Percentage points were shown as 100 stylised human figures. We colour those in traffic light colours to indicate improvement (green), no change (yellow), and deterioration (red), and similarly for postoperative problems (no/some/extreme).

To abstract from the concept of probability, we introduce each graph with the text *“This is how 100 patients like you felt after surgery”*. This phrase helps patients to put the presented amounts into context and also emphasises the aspect of risk stratification. Proportions were rounded so that they always sum to 1 (100%). Results are presented in terms of overall impact on health and for each of the EQ-5D dimensions.

## Results

### Risk stratification

Our development sample consisted of 497,723 patients with complete pre- and postoperative EQ-5D-3L health profiles and no missing information on any of the relevant risk variables.^3^ The descriptive statistics for the development sample are reported in Table 1. For all three treatments, the patient populations’ pre-operative HRQoL spanned more than 160 EQ-5D-3L health profiles, thereby covering a large proportion of the 243 (=3^5^) possible EQ-5D-3L health profiles. This variability facilitates the identification of interaction effects between health dimensions. For comparison, a representative sample (*n* = 7294) of the general population in England reported 98 unique EQ-5D-3L health profiles [7], and participants in a multi-country instrument validation study drawn from eight patient groups and a student cohort (*n* = 3919) described their HRQoL using 124 unique EQ-5D-3L health profiles [16]. Despite the wide coverage, the distribution of health profiles in our sample is highly concentrated, as is observed in other studies using the EQ-5D-3L [7]. More than 90% of patients in each of the three treatment groups could be described by no more than 17 profiles.

**Table 1:** Descriptive statistics of development sample

	Hip replacement (*N* = 185,111)		Knee replacement (*N* = 198,007)		Groin hernia repair (*N* = 114,605)	
Age groups (*n*, %)						
15–29	328	0.2%	12	0.0%	2426	2.1%
30–39	1139	0.6%	146	0.1%	4803	4.2%
40–49	6022	3.3%	2319	1.2%	12,191	10.6%
50–59	24,579	13.3%	21,765	11.0%	20,660	18.0%
60–69	62,871	34.0%	72,153	36.4%	36,618	32.0%
70–79	67,079	36.2%	76,997	38.9%	28,280	24.7%
80–89	22,419	12.1%	24,169	12.2%	9287	8.1%
≥90	674	0.4%	446	0.2%	340	0.3%
Gender (*n*, %)						
Female	109,892	59.4%	112,019	56.6%	6230	5.4%
Male	75,219	40.6%	85,988	43.4%	108,375	94.6%
Symptomperiod (*n*, %)						
<1 year	25,831	14.0%	9,863	5.0%	74,896	65.4%
1–5 years	127,008	68.6%	103,841	52.4%	39,709	34.6%
6–10 years	20,386	11.0%	43,308	21.9%		
>10 years	11,886	6.4%	40,995	20.7%		
Pre-operative EQ–5D						
Utility score (mean, SD)	0.356	0.319	0.414	0.309	0.791	0.196
Profile—MO (*n*, %)						
1	12,299	6.6%	13,553	6.8%	92,640	80.8%
2	172,278	93.1%	184,053	93.0%	21,907	19.1%
3	534	0.3%	401	0.2%	58	0.1%
Profile— SC (*n*, %)						
1	84,533	45.7%	138,356	69.9%	110,629	96.5%
2	98,739	53.3%	58,391	29.5%	3815	3.3%
3	1839	1.0%	1260	0.6%	161	0.1%
Profile—UA (*n*, %)						
1	11,054	6.0%	18,467	9.3%	83,597	72.9%
2	140,344	75.8%	155,240	78.4%	28,829	25.2%
3	33,713	18.2%	24,300	12.3%	2179	1.9%
Profile—PD (*n*, %)						
1	1,275	0.7%	1,837	0.9%	37,014	32.3%
2	106,670	57.6%	120,539	60.9%	72,975	63.7%
3	77,166	41.7%	75,631	38.2%	4616	4.0%
Profile—AD (*n*, %)						
1	109,184	59.0%	125,807	63.5%	97,287	84.9%
2	67,642	36.5%	65,184	32.9%	16,296	14.2%
3	8285	4.5%	7016	3.5%	1022	0.9%
Postoperative EQ–5D						
Utility score (mean, SD)	0.785	0.246	0.724	0.257	0.876	0.189
Patients' overall assessment of outcome (*n*, %)						
Improved	149,127	80.6%	141,273	71.3%	54,767	47.8%
No change	29,775	16.1%	44,420	22.4%	43,771	38.2%
Deteriorated	6209	3.4%	12,314	6.2%	16,067	14.0%

**Fig. 1:**
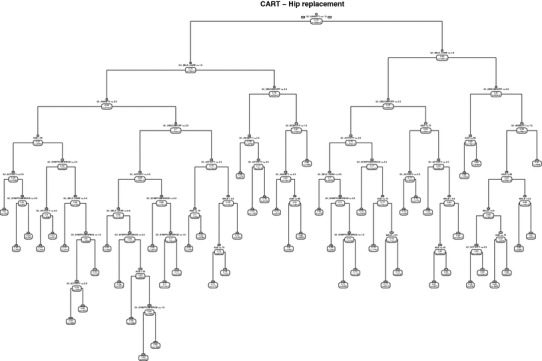
Regression tree for hip replacement. Branches for pre-operative EQ-5D health profiles 11111 and 33333 not shown

The regression trees classified patients into 55 (hip replacement), 59 (knee replacement) and 60 (groin hernia repair) distinct groups (Table 2). Figure 1 shows as an example the tree structure for hip replacement surgery. The groups in each tree were well populated, with median group sizes of 1732 (IQR=674–6182) for hip replacement, 1269 (IQR=474–4337) for knee replacement, and 564 (IQR=240–2018) for groin hernia repair. These groups explained 14–27% of the variance in postoperative EQ-5D utility scores in the development sample, with similar, albeit slightly attenuated performance in the test sample. Conversely, a model based on age, sex and symptom period (‘reduced model’) explains no more than 2% of the variance.

**Table 2:** Predictive performance of risk stratification algorithm

Procedure	#groups	Development sample		Test sample		Reduced model	
		*adj·R* ^2^	RMSE	*adj·R* ^2^	RMSE	*adj·R* ^2^	RMSE
Hip replacement	55	14.3%	0.228	12.8%	0.218	1.5%	0.244
Knee replacement	59	19.4%	0.231	18.8%	0.224	2.1%	0.255
Groin hernia repair	60	27.0%	0.161	28.1%	0.158	1.3%	0.188

Development sample: April 2009 to March 2015. Test sample: April 2015 to March 2016. Reduced model only considers age, sex and symptom period for grouping and is estimated and tested on the development sample. *R* ^2^ is adjusted for number of predictor variables, i.e. groups

The MIDs for improvements/deteriorations are reported in Table 3. MIDs for hip and knee replacement are similar in magnitude. Improvements need to be larger to be noticeable to patients than deteriorations, i.e. the MIDs are not symmetric. Estimates for groin hernia repair are substantially different.

**Table 3:** Estimates of minimally important differences (MIDs)

Procedure	MID - Improvement		MID - Deterioration	
	Est	95% CI	Est	95% CI
Hip replacement	0.106	(0.095–0.116)	−0.091	(−0.075 to −0.106)
Knee replacement	0.090	(0.083–0.097)	−0.081	(−0.071 to −0.090)
Groin hernia repair	0.041	(0.033–0.048)	−0.069	(−0.056 to −0.081)

**Fig. 2:**
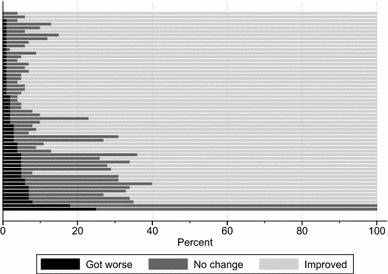
Differences in proportion of hip replacement patients reporting significant improvements, deteriorations or no change across 55 risk strata (nodes)

Figure 2 illustrates the importance of risk stratification for the purposes of classifying hip replacement patients according to their probability of improving, deteriorating or not experiencing any noticeable change in their HRQoL. Each stacked horizontal bar represents these probabilities for one of the 55 risk groups. There is marked variation in predicted outcomes across groups, with twelve groups (*n* = 52,850 patients) showing <70% risk of improvement and thirteen groups (*n* = 39,883) showing ≥95% risk of improvement (based on rounded numbers). It is also instructive to compare these to a prediction for the average patient in the sample as would often be presented in existing decision aids. The average patient has an 81% risk of improvement (and a 3% risk of deterioration)(see Table 1). Only two groups, representing a total of *n* = 12,076 patients, have a predicted risk of improvement of ±5% around this average. Hence, for the vast majority of patients, information about the average risk of improvement would likely be misleading.

### Online tool user interface

**Fig. 3:**
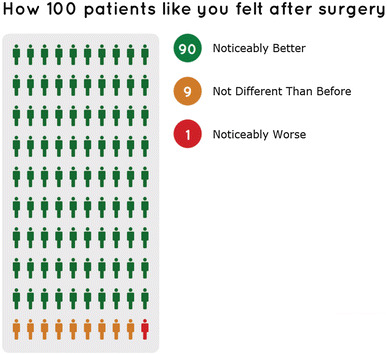
Screenshots of the user interface

Figure 3 gives examples of the feedback that patients receive after having provided information on their pre-operative HRQoL, age, gender and symptom period. Patients will first be presented with information on the proportion of patients achieving a minimally important difference. They can then request detailed information on the predicted postoperative HRQoL in a similar format, print the results, or amend the information they provided. In all cases, patients are urged to discuss the results with their GP before making a decision. They are also reminded that the results are based on a snapshot of their HRQoL on that day and may therefore change over time as their HRQoL (or the reporting thereof) changes.

The online tool has been designed following best practice for maximising accessibility. It has been tested by local GPs in York (United Kingdom), members of the Vale of York Clinical Commissioning Group, a patient representative and a prospective patient, and two vision impaired members of staff. This process led to changes in wording and colour scheme, and a reduction in the number of patient characteristics considered for risk stratification (see Section 2.2). The overall feedback indicates that the webtool is easy to use and that the presentational format aids understanding of the information provided.

## Discussion

Informing prospective patients about the likely outcomes of treatment as part of SDM can help shape realistic expectations, improve satisfaction with treatment choices and outcomes, reduce decision uncertainty and may reduce demand for major invasive surgery [27]. But the information that most doctors can relay is limited to the *average* outcome experienced by patients in clinical trials. For many patients, this will be an inaccurate or even misleading reflection of their likely outcome, either because the clinical trials did not sample similar patients or because their personal characteristics and, hence, likely outcomes are substantially different from the average person enrolled in the trial.

There is an increasing policy push towards routine collection of PROM data to improve healthcare delivery in a number of health systems including Sweden, Australia, Canada, the Netherlands, the USA and the UK. The advent of large-scale data collection of the experiences of patients treated in routine practice makes it possible to develop risk stratification algorithms and provide patients with information that more closely reflects their individual circumstances. But this information needs to be presented in an accessible and understandable fashion in order to support SDM between patients and doctors. In this paper, we have demonstrated a method for presenting information about the effectiveness of treatment according to the specific characteristics of prospective patients, rather than in terms merely of average effects. We have also shown how the information can be made available to patients and doctors in an interactive format to help support SDM.

The multidimensional nature of HRQoL presents some unique challenges in developing a patient information tool. Prospective patients are likely to differ in the amount of information they can process effectively. Some patients will prefer a simple summary of the likely outcomes they may experience such as the MID. Others may wish to see predictions by HRQoL. To ensure that the underlying stratification is consistent across both presentational formats, we decided to group patients according to their postoperative EQ-5D utility scores and then translate that information into MIDs but also allow retrieval of the underlying EQ-5D health profiles. There is some evidence that the relationship between patient characteristics and outcome differs by EQ-5D dimension [9], so that dimension-specific stratification algorithms might generate different, more accurate, groupings than that developed on EQ-5D utility scores. McCarthy [19, 20] has recently suggested a two-step approach to combine separate treatment effect estimates by EQ-5D domain into a composite effect. It may be possible to extend this methodology to risk stratification, something that might merit further exploration.

Our current stratification algorithms explain from 14% (hip replacement) to 27% (hernia repair) of variation in EQ-5D utility scores three or six months after surgery. A similar algorithm developed to predict EQ-5D utility scores in a large Swedish hip replacement population one year after surgery was able to explain 17% of variation [21]. Performance may be enhanced by stratifying on a larger number of patient characteristics, although these gains in explanatory power need to be balanced against reduced usability during time-constraint GP consultations, as more time would be required to complete the interface entry. Perfect explanatory power is an unrealistic ambition, with a substantial part of the variation in HRQoL likely to remain unexplained because it either originates from random statistical variation or reflects patient characteristics that are impossible to observe prior to surgery such as the patient’s future adherence to the postoperative recovery plan [28]. Even with limited explanatory power, prospective patients will still benefit from receiving tailored predictions instead of information on average outcomes.

There are a number of ways in which this work can be taken forward. The current version of the online tool is informative only about the outcome of surgery but does not provide information on what would have happened in its absence, i.e. under watchful waiting or other forms of treatment. We are aware of some local initiatives to collect such data in Gloucestershire, UK and Alberta, Canada. These initiatives offer the prospect of providing information about alternative courses of treatment so that, in future, patients can be informed by comparative assessments.

A second issue arises from the use of patient-reported data to stratify risk groups. These data are likely to vary over measurement occasions, and so, for example, a patient may report some pain and discomfort on Monday and extreme levels on Tuesday. This implies that the information presented is conditional on how they are feeling at the time and, consequently, their predicted outcomes may vary as well. There are two solutions. One is to collect self-assessed HRQoL longitudinally to better isolate true level of HRQoL from random variation. The other is to ignore self-assessed data and use only objective data (such as age and gender), but this comes at the expense of explanatory power.

Finally, personalised medicine can be understood to involve not only risk stratification but also approaches to incorporating preference heterogeneity amongst patients [26]. We currently base all calculations on EQ-5D index scores derived using the MVH-A1 tariff [6]. But value sets are not neutral and the choice of valuations has important effects on the distribution of EQ-5D index scores and any inferences based upon them [22]. Previous research has shown that value sets derived from specific patient populations differ systematically from those derived from the general population [18], and it is likely that even within patient groups, there exists substantial heterogeneity in preferences. However, eliciting preferences from individual patients, as sometimes done in SDM, would also require deriving individual measures of MIDs to fit with our current presentational format and this may be difficult for patients to determine prior to surgery.

In conclusion, we believe that large administrative PROM datasets offer the opportunity to derive individualised predictions of the likely outcome of treatment, thereby helping patients to make better decisions, generate more realistic expectations about treatment outcomes, and increase satisfaction with treatment.

## Footnotes

1 We did not include varicose vein patients since the number of complete data points is substantially lower and a large number of patients report pre-operative EQ-5D-3L health profiles as 11111, i.e. there is no capacity to improve.

2 In doing so, we generated an update to their MID estimates obtained from a much smaller sample.

3 In some cases, missing information was collected but not released by the HSCIC as part of their publicly available dataset to ensure that patients cannot be re-identified. See also FN2.
